# Influence of the Plantar Cutaneous Information in Postural Regulation Depending on the Age and the Physical Activity Status

**DOI:** 10.3389/fnhum.2016.00409

**Published:** 2016-08-17

**Authors:** Julien Maitre, Thierry P. Paillard

**Affiliations:** Laboratoire Mouvement Equilibre, Performance et Santé, EA 4445, Département Sciences et Techniques des Activités Physiques et Sportives (STAPS), Université de Pau et des Pays de l’AdourTarbes, France

**Keywords:** balance, aging, physical activity, foam, cutaneous, postural control

## Abstract

The aim was to compare the balance control adaptation to different supporting surfaces depending on the age and the physical activity status. The balance control of two groups of young (*n* = 17) and old (*n* = 17) participants who practiced regular physical activity (active groups) and two groups of young (*n* = 17) and old (*n* = 17) participants who did not practice physical activity (non-active groups) was compared on a firm surface and on a foam surface. The parameters of the center of foot pressure (COP) displacement were compared between the groups. The two older groups were more disturbed than the two younger groups when they stood on a foam surface and there was no difference between active and non-active groups. This result may be linked to the structural and functional involutions of the plantar cutaneous sole and foot that occur with age advancement. The participants’ physical activity practice might be not specific enough to generate a more efficient postural adaption to the foam condition for the active groups than the non-active groups within their respective age groups.

## Introduction

The skin is a highly complex interface, innervated by a wide array of specialized sensory neurons sensitive to heat, cold, pressure, irritation, itch and pain (McGlone and Reilly, 2010). Tactile information provides feedback about the environment that contributes to balance control (Massion, 1994; Palluel et al., 2008). Indeed, as the feet interface directly with the ground, the cutaneous afferents, emanating from the soles of the feet, provide sensory information on force distribution during upright stance. Thereby, the plantar sole may be considered as a “dynamo-metric map” (Kavounoudias et al., 1998). To maintain an upright stance, the central nervous system (CNS) integrates cutaneous afferents with other sensory afferents emanating from visual, vestibular and proprioceptive systems (Massion, 1994). An efficient balance control requires availability and accuracy of the sensory afferents (Maitre et al., 2013a). In the context of exogenous perturbation that alters postural segment positions and compromise upright stance, the CNS triggers compensatory postural strategies (Horak and Nashner, 1986) and sensory reweighting (Oie et al., 2002) to preserve balance. Nevertheless, with increasing age, individuals undergo involutions, which result in balance disorder and reduce the ability to compensate for unreliable or discordant sensory input (Sturnieks et al., 2008). These involutions may decrease the efficiency of the central processing mechanisms (Hay et al., 1996) and the neuromuscular function (Aagaard et al., 2010). Moreover, the functional ability of the sensory systems may be reduced with increasing age (Sturnieks et al., 2008).

Although aging has deleterious effects on balance control, the iterative stimulations of the visual, vestibular and proprioceptive systems, induced by the regular practice of physical activity, are known to preserve their functional abilities (Gauchard et al., 2001, 2003; Ribeiro and Oliveira, 2007) and can even improve their contribution in the postural regulation (Quarck and Denise, 2005; Jafarzadehpur et al., 2007; Aman et al., 2015). Moreover, the beneficial effects of the physical activity can also contribute to enhance the ability to detect the plantar pressure distributions (Schlee et al., 2007; Li and Manor, 2010).

It is known that foot problems may occur with aging and are associated with impaired balance and functional ability and increased risk of falls (Menz et al., 2006). With increasing age, the foot undergoes structural and functional involutions, which may result in flatter feet, reduced range of motion of the ankle joint, a higher prevalence of hallux valgus, toe deformities and toe plantarflexor weakness, and reduced plantar tactile sensitivity (Scott et al., 2007). Since the physical activity appears to have opposite effects to the age-related involutions in terms of balance control, it would be interesting to clarify the resultant between the benefits induced by the physical activity and the involutions induced by aging in an ecological context. The use of a foam-supporting surface appears to be a relevant tool to challenge balance control and produces a substantial and multi-directional balance perturbation (Patel et al., 2008a,b) to detect age-related changes (Choy et al., 2003) and exercise (Hue et al., 2004) effects on the postural function. Static standing on a foam surface would change the multiple biomechanical variables in the foot, resulting in an alteration of the distribution of the plantar pressures (Chiang and Wu, 1997). Consequently, the aim of this study was to compare the balance control adaptation to different characteristics of the supporting surface (i.e., firm surface and foam surface) between young and old participants in relation to their physical activity status (i.e., active and non-active). We hypothesized that physically active participants would demonstrate better postural control in context of an altered support surface (i.e., foam surface) than non-active participants whatever the age considered.

## Materials and Methods

### Participants

Recruitment for this study involved the participation of 68 women who gave their informed consent. The experiments received the approval of the local committee for the protection of human participants. All the participants were free from any disorder after medical examination (i.e., neurological, motor and metabolic disorders). The cutaneous sensations under the feet were screened with a pencil and participants were free from any foot disorders and lesion of the foot skin support surface. Four groups were made up according to the age (i.e., young and old) and the physical activity status (i.e., active and non-active). The inclusion criteria in each group were previously described (Maitre et al., 2013b). The young active group (*n* = 17; age: 20.5 ± 1.1 years; height: 164.8 ± 5.7 cm; weight: 60.5 ± 7.1 Kg; foot size: 26.0 ± 0.9 cm) was formed with sports science students who have practiced sports (for 3 h or more each week, at least at regional level; i.e., swimming, gymnastics, handball, basketball, athletics). The young non-active group (*n* = 17; age: 20.0 ± 1.3 years; height: 162.3 ± 5.4 cm; weight: 56.2 ± 9.2 Kg; foot size: 25.5 ± 0.8 cm) was formed with students who have not practiced physical activities for at least 3 years. The old active group (*n* = 17, age: 74.0 ± 3.8 years; height: 156.6 ± 4.2 cm; weight: 63.2 ± 6.9 Kg; foot size: 25.6 ± 0.8 cm) was formed with healthy older women who practice (for 3 h or more each week) and have regularly practiced (for at least 3 years) physical activity in a sports club (i.e., gymnastics, walking, dancing, aquarobics). The old non-active group (*n* = 17, age: 74.7 ± 6.3 years; height: 155.8 ± 5.7 cm; weight: 62.4 ± 9.0 Kg; foot size: 25.3 ± 0.7 cm) was formed with healthy older women who have not practiced any physical activity (for at least 3 years) except for daily tasks.

### Measurements

The participants were tested on a force platform with three strain gauges (Techno Concept^TM^ Mane, France; 40 Hz frequency, 12 bit A/D conversion) in two bipodal conditions with eyes open (i.e., eyes fixed straight ahead on a target at 1.5 m). They were asked to take an upright stance (i.e., barefoot, 30° of feet angle, inter-malleolar distance of 9 cm), as still as possible, with their arms at their side in a “reference condition” (REF condition, i.e., stance on a firm surface) and in a “Foam condition”. During the Foam condition, the participants were asked to take an upright stance on a foam surface (15 mm, 70 kg.m^−3^; TG700, Domyos^®^, Villeneuve d’Ascq, France) fitted on the force platform to modify the contribution of plantar cutaneous information in postural regulation (Chiang and Wu, 1997). The main objective of the foam condition was to alter plantar cutaneous sensory information in order to determine the reliance attributed to this input in postural regulation. Each condition lasted 20 s. Before the two condition tests, participants benefited from at least 2 min of familiarization with the force platform (as still as possible with eyes open and eyes closed).

The acquisition of the center of foot pressure (COP) displacements was done using Posturowin software (Techno Concept^TM^, Mane, France) to calculate the parameters that give features of the balance control: the postural stability (i.e., COP surface, mm^2^; Caron et al., 2000) and the postural control (i.e., the COP velocity, mm.s^−1^; Caron et al., 2000). The COP velocity may be calculated for the frontal (COP_X_ velocity, mm.s^−1^) and the sagittal (COP_Y_ velocity, mm.s^−1^) directions. The smaller these parameters (i.e., velocity and surface) the better the balance control.

### Statistical Analysis

Statistical treatment of data was achieved using Statistica software. The one-factor analysis of variance (ANOVA) was used to test the mean differences among the four groups for the anthropometrical data and for the COP displacement parameters in the reference condition. A three factor ANOVA with repeated measures was used to test the condition (reference and foam condition), the age (young and old) and the activity (active and non-active) effects. The differences among means were tested using the Newman-Keuls *post hoc* analysis when significant main effect was found. The results were considered significant at the level of 5%.

## Results

### Age and Anthropometrical Data

The results indicated significant age differences between the younger and older groups (*F* = 1158.7, *p* < 0.001). The young active and non-active groups were respectively younger than the old active (*p* < 0.001 and *p* < 0.001) and non-active (*p* < 0.001 and *p* < 0.001). Furthermore, there were significant height differences between the younger participant groups and the older participant groups (*F* = 11.8, *p* < 0.001). The young active and non-active groups were respectively taller than the old active (*p* < 0.001 and *p* < 0.01) and non-active (*p* < 0.001 and *p* < 0.01).

### Center of Foot Pressure Displacement Parameters

**Figure 1:**
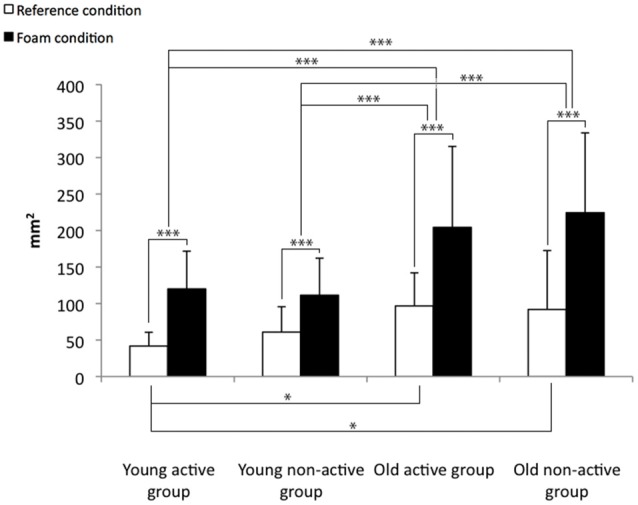
**Means and standard deviations for the center of foot pressure (COP) surface for the reference and the foam conditions.** *p < 0.05, ***p < 0.001.

**Figure 2:**
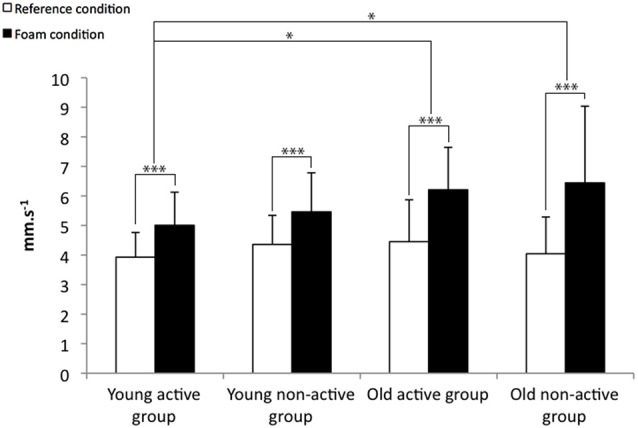
**Means and standard deviations for the COP_X_ velocity for the reference and the foam conditions.** *p < 0.05, ***p < 0.001.

**Figure 3:**
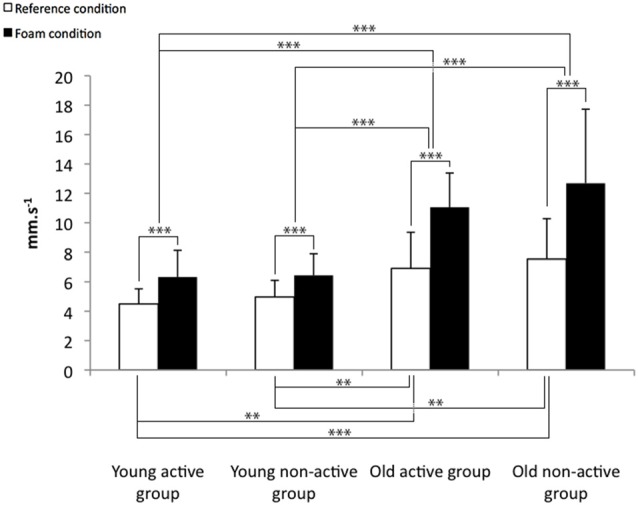
**Means and standard deviations for the COP_Y_ velocity for the reference and the foam conditions.** **p < 0.01, ***p < 0.001.

The COP displacement parameters for the reference and the foam condition is presented in the Figures 1–3.

#### Reference Condition

The results concerning the reference condition comparisons indicated that the COP surface (*F* = 4.6, *p* < 0.01) and the COP_Y_ velocity (*F* = 9.3, *p* < 0.001) differed significantly between the four groups. The *post hoc* analyses indicate that the COP surface (Figure 1) and the COP_Y_ velocity (Figure 3) were lower for the young active group than for the two older groups (i.e., the active and non-active groups) in the reference condition. Furthermore, the COP_Y_ velocity (Figure 3) was lower for the young non-active group than the two older groups (i.e., the active and non-active groups).

#### Differences Between the Firm and Foam Condition

All the COP displacement parameters presented significant condition effects for the COP surface (*F* = 82.4, *p* < 0.001), the COP_X_ velocity (*F* = 77.5, *p* < 0.001) and the COP_Y_ velocity (*F* = 110.4, *p* < 0.001). These results indicate that the COP displacement parameters increased for all the groups (Figures 1–3). Otherwise, there is a significant condition × age interaction for the COP surface (*F* = 7.5, *p* < 0.01), the COP_X_ velocity (*F* = 7.5, *p* < 0.01) and the COP_Y_ velocity (*F* = 25.0, *p* < 0.001). These results indicate that the balance control was more altered for the older participants than for the younger participants, particularly on the Y-axis. The COP surface and the COP_Y_ velocity increased less for the two younger groups (i.e., the active and non-active) than for the two older groups (i.e., the active and non-active; Figures 1, 3). In addition, the COP_X_ velocity increased less for the young active group than the two older groups (i.e., the active and non-active groups; Figure 2).

## Discussion

The aim of this study was to compare the balance control adaptation to different characteristics of the supporting surface (i.e., firm surface and foam surface) between young and old participants in relation to their physical activity status (i.e., active and non-active). We did not find support for our hypothesis. The main result of this study indicated that there is no difference between active and non-active participants within their respective age groups for the reference and the foam conditions. The older participants demonstrated less efficient balance control on the firm surface (i.e., reference condition) than the younger participants. Moreover, balance control was disturbed for all the participants when they stood on a foam surface, but the older participants were more disturbed than the younger participants.

As suggested by previous results, aging alters balance control even for a simple postural task (i.e., bipedal stance with eyes open on a firm surface; Choy et al., 2003). In the present study, the COP_Y_ velocity differences between the younger and older groups indicated that older participants demonstrated a poorer postural control than the younger participants, in the anteroposterior direction. This result is supported by the literature (Du Pasquier et al., 2003) and may be due to the unavoidable involutions of the postural function (i.e., alteration of the sensory, central processing, and muscular functions; Sturnieks et al., 2008). Furthermore, the postural differences between younger and older individuals may be more highlighted in the context of challenged postural task (Maitre et al., 2013a,b).

In the present study, when participants stood on the foam surface the COP surface and the COP velocities on the X and Y axes increase significantly as compared to the firm surface. The disturbing effects of standing on a foam surface have been investigated previously in several studies (Chiang and Wu, 1997; Patel et al., 2008a,b). Static standing on a foam surface would change the multiple biomechanical variables in the foot, resulting in an alteration of the distribution of the plantar pressures (Chiang and Wu, 1997). Foam condition would affect the input of both joint and cutaneous mechanoreceptors. The foam condition would alter the spatial configuration of the cutaneous mechanoreceptors in the foot, thereby reducing the ability to sense pressure distribution and body orientation (Chiang and Wu, 1997). Moreover, the mechanical properties of the compliant surface may also alter the postural behavior. The elastic characteristics of the foam enable smaller opposing mechanical resistance than a firm surface that could cushion the foot movements produced by the ankle musculature, which reduces the motor output generated for postural stabilization (Horak and Hlavacka, 2001; Patel et al., 2008b). Consequently, the balance control is altered on a foam-supporting surface.

Relative to the reference condition, the COP surface and the COP_Y_ velocity increased significantly more for the older groups than the younger groups while standing on the foam. This result corroborates previous work (Choy et al., 2003), which indicated that older participants demonstrated poorer balance control on a foam-supporting surface than younger participants. The poorer postural control achieved by the older groups compared to the younger groups could be explained in two ways. Firstly, it could be linked to a plantar tactile sensitivity malfunction in the older groups in comparison with the younger groups. Although participants of this study were free from any foot disorders and lesion of the foot skin support surface, we cannot exclude that the plantar tactile sensitivity differs between the younger and older groups. Indeed, it is known that foot and ankle undergo involutions with aging that may alter balance function (Menz et al., 2006). Older individuals may exhibit flatter feet, reduced range of motion of the ankle joint, a higher prevalence of hallux valgus, toe deformities and toe plantarflexor weakness, and reduced plantar tactile sensitivity (Perry, 2006; Scott et al., 2007). Cutaneous mechanoreceptors decrease in number and have a progressive structural deterioration with ageing (Shaffer and Harrison, 2007; Decorps et al., 2014). These elements may contribute to decrease the ability of the older groups to accurately detect foot pressure distribution and correctly regulate posture (Menz et al., 2006). Indeed, several authors (Simmons et al., 1997; Horak and Hlavacka, 2001) showed that when somatosensory information is reduced postural control might be altered**.** Secondly, the poorer postural control achieved by the older groups compared to the younger groups could be linked to the inability of the older groups to take advantage of the non-altered sensory information as effectively as the younger groups. Indeed, in a context of a sensory alteration, the balance control mechanism requires the availability as well as the accuracy of information emanating from the non-altered sensory systems to enable the CNS to integrate the information and to initiate a compensatory motor strategy limiting the postural disturbance related to the foam surface (Massion, 1994; Peterka, 2002). When cutaneous information is reduced, individuals are compelled to rely more on other sensory systems (i.e., visual, vestibular and proprioceptive systems) to maintain their stability (Lord and Menz, 2000; Horak and Hlavacka, 2001; Paillard et al., 2007). Although, Meyer et al. (2004) suggested that, in a context of plantar cutaneous anesthesia, plantar sensation is of moderate importance for the maintenance of normal standing balance, the impact of reduced plantar sensitivity on postural control increases with the loss of additional sensory modalities. With increased age, there is a progressive loss of functioning of visual, vestibular and proprioceptive systems, which can contribute to balance deficits (Sturnieks et al., 2008). In addition, the ability to correctly reweight sensory information may be altered with aging (Horak et al., 1989). Thereby, in the present study, the older participants may be less able to counteract postural disruption due to the foam-supporting surface than the younger groups.

The main result indicated that there is no difference between active and non-active participants within their respective age groups for the reference and foam conditions, yet, previous studies have demonstrated that regular practice of physical activity may enhance balance control for young (Kiers et al., 2013) and old (Howe et al., 2007) individuals. In the present study, several elements could explain this lack of significant results. Firstly, as suggested by Kiers et al. (2013), the reference condition (i.e., bipedal stance with eyes open on a firm surface) probably constitutes a too simple postural task to produce evidence of differences between active and non-active participants. Secondly, the active participants might have improved their ability to maintain dynamic balance more than static balance with their physical practice. Indeed, the ability to maintain balance is likely to be specific to the training tasks (Hrysomallis, 2011). In this study, the postural tasks were statics and the active participants mainly practiced dynamic physical activities. Thirdly, the rubber of foam used in the foam condition was probably too thin to sufficiently challenge the balance control in order to demonstrate difference in relation to the physical activity status. Patel et al. (2008a,b) have suggested that thin rubber of foam enables closer contact with the rigid surface beneath the foam, which allows plantar tactile sensory feedback and ankle movements to be more effective. In addition, the deformation properties of the foam surface matched to the participants’ weight (Gosselin and Fagan, 2015). The heavier participants might be in closer contact with surface beneath the foam. In the present study, the foam characteristics were relevant to highlight differences in relation to the age, but probably not appropriate to highlight difference in relation to the physical activity status. Caution should be taken when using foam pads on force platforms during balance assessments. To evidence a postural control difference in relation to the physical activity status, the postural task difficulty has to be accurately calibrated, since the foam properties (Patel et al., 2008a,b) and the anthropometric characteristics of the participant (Gosselin and Fagan, 2015) may alter posturographic data.

This study suggests that the ability to detect the distribution of the plantar pressure contributes to the balance control. Although foot problems may occur with increasing age (Menz et al., 2006), previous studies have demonstrated that augmenting tactile sensory information from the sole of the foot may improve balance in older people (Palluel et al., 2008). Furthermore, the regular practice of physical activity may improve the tactile sensitivity (Schlee et al., 2007; Kerr et al., 2008; Li and Manor, 2010; Kattenstroth et al., 2013). Nevertheless, in the present study, the effect of the regular practice of physical activity appeared to be not strong enough and/or appropriate to generate postural difference between active and non-active participants within their respective age groups. It is known that physical activity induces specific postural adaptation (Hrysomallis, 2011). In the present study, it could be construed that the characteristics of the physical activities practiced by the participants might not be specific enough to generate a more efficient postural adaption to the foam condition for the active groups than the non-active groups within their respective age groups (Paillard et al., 2010). Most of the participants in the active groups do not practice barefoot physical activities that specifically stimulate cutaneous mechanoreceptors. Specific training that stimulates plantar cutaneous sensory mechanoreceptors improves postural function (Morioka et al., 2011). Practiced barefoot, physical activity could enhance plantar cutaneous sensitivity (Schlee et al., 2007). Hence, the data of the present study suggest that the practice of barefoot exercises could be recommended to improve efficiency of the postural function on altered support surface.

## Conclusion

This study corroborates that, with increased age, there is an alteration of the contribution of plantar cutaneous information. This loss of functioning was present for a simple postural task (i.e., upright stance with eyes open on a firm surface) and for a more complex postural task (i.e., upright stance with eyes open on a foam surface). Relative to the reference condition, when the cutaneous sensory information was altered by the use of a foam-supporting surface, the balance control was more disrupted for the older participants than the younger participants. The inability to counteract this sensory alteration might be linked to the unavoidable structural and functional involutions of the plantar cutaneous sole and foot that occur with age advancement. The main result of this study suggests that there is no difference between active and non-active participants within their respective age groups in the foam conditions. Physical activity traditionally practiced with shoes might not sufficiently stimulate the cutaneous mechanoreceptor to counteract the effects of aging on the plantar skin sensitivity. In order to extend the analysis of the effects of physical activity on the cutaneous sensitivity in relation to the age and the physical activity status, participants should be compared according to the type of sports (i.e., with shoes or barefoot) and the cutaneous sensitivity should be analyzed by the use of specific tests (e.g., vibrotactile or monofilament sensitivity**)**. Identifying influence of barefoot physical activity would enable clinician to prevent loss of plantar skin sensitivity and so improve postural control in older people.

## Author Contributions

JM and TPP contributed to the conception and design of the work; and the acquisition, analysis, and interpretation of data.

## Funding

The investigation was supported by grants from the Association Nationale de la Recherche et de la Technologie and the Conseil Général des Hautes Pyrénées.

## Conflict of Interest Statement

The authors declare that the research was conducted in the absence of any commercial or financial relationships that could be construed as a potential conflict of interest.
